# Supplementary material for: MetaRibo-Seq measures translation in microbiomes
Source: Nat Commun. 2020 Jun 29;11:3268. doi: 10.1038/s41467-020-17081-z (PMC7324362; doi:10.1038/s41467-020-17081-z)
Supplement: Supplementary file 10 — Supplementary Data 7 [file 41467_2020_17081_MOESM10_ESM.zip › File2/Confidence_High_Taxonomy/326709_out.krona.html]

Javascript must be enabled to view this page.

members
magnitude
magnitudeUnassigned
count
unassigned
taxon
rank

326709\_out

2

2
superkingdom
2

2
phylum
976

class
2
200643

2
order
171549

family
2
171552

838
2
genus

species
2

SRS049959\_contig\_number\_52544SRS144537\_contig\_number\_5379
1262925
